# The Enigma of Autoimmunity: A Case of Subacute Sclerosing Panencephalitis in a Patient Diagnosed With Systemic Lupus Erythematosus

**DOI:** 10.7759/cureus.37602

**Published:** 2023-04-15

**Authors:** Al Inde John A Pajantoy, Lester D Dimzon, Pia Teresa A Camara, Amado M San Luis

**Affiliations:** 1 Department of Adult Neurology, Center for Neurological Sciences, Quirino Memorial Medical Center, Quezon City, PHL; 2 Department of Clinical Neurosciences, University of the East Ramon Magsaysay Memorial Medical Center, Quezon City, PHL

**Keywords:** autoimmune, dyken’s criteria, encephalitis, measles, sle, sspe

## Abstract

Subacute sclerosing panencephalitis (SSPE) is a rare complication of measles characterized by progressive neurological deterioration. The onset usually occurs about seven to 10 years after the measles infection. Aside from an earlier age of measles infection, factors that may influence the susceptibility for its development is unknown. There is a paucity of data regarding the course of SSPE in the presence of concomitant autoimmune conditions such as systemic lupus erythematosus (SLE). We report a case of a 19-year-old female who presented with new onset recurrent generalized tonic-clonic seizures, malar rash, and cutaneous erythematous, maculopapular eruptions. Antinuclear antibodies (ANA) and anti-double stranded DNA (anti-dsDNA) serologic examination yielded positive results favoring the diagnosis of SLE. Further in the course of illness, the patient developed generalized myoclonic jerks and progressive decline in language, cognitive, and motor functions. Subsequent investigation showed an elevated anti-measles antibody titer in the cerebrospinal fluid, and periodic generalized, bilaterally synchronous, and symmetric high voltage slow-wave complexes in the EEG. These findings and the typical evolution of neurologic manifestations fulfilled two major and one minor Dyken’s criteria for the diagnosis of SSPE.

It is postulated that some autoimmune-mediated responses may contribute to the evolution of SSPE. Autoimmune complexes in SLE induce downregulation of T-cell responses which accelerate the loss of antibodies formed against other diseases such as the measles virus that may lead to increased susceptibility to infection. SSPE is hypothesized to result from the downregulation of host-immune responses which leads to incomplete measles viral clearance. To the best of the authors’ knowledge, this is the first published case of SSPE with active SLE.

## Introduction

Subacute sclerosing panencephalitis (SSPE) is recognized as a result of chronic measles infection. The condition is characterized as a progressive neurological deterioration affecting cognition and behavior, development of seizures, and eventual vegetative state [1]. According to the World Health Organization, the true incidence of SSPE is approximately four to 11 cases per 100,000 cases of measles [2]. The incidence of the disease has fallen sharply due to vaccination programs [1]. Epidemiological studies done in the Philippines from 1999 to 2006, revealed that there are only 60 well-documented patients identified to have SSPE. Fifty-five (55%) percent of these individuals were from the National Capital Region with a male preponderance and a mean age of 9.8 years at the time of diagnosis [3]. An earlier age of onset increases the risk for its development [4]. Concomitant human immunodeficiency virus (HIV) infection may place a higher risk of developing SSPE along with a more aggressive course and earlier onset has been described [5]. There is a paucity of data regarding possible association on how latent measles infection might be linked to the development of systemic lupus erythematosus (SLE), and on the other spectrum, whether concomitant autoimmune conditions such as SLE can influence the development of clinical manifestations of SSPE. To the best of the authors’ knowledge, no other published reports on an individual diagnosed with concomitant SLE and SSPE have been made at the time of writing.

This article was previously presented at the 18th Asian Oceanian Congress of Neurology (AOCN) and the 29th Annual Conference of the Indian Academy of Neurology (IANCON) on November 3 to 6, 2022 as an electronic poster.

## Case presentation

A 19-year-old, female, of Filipino descent, born of a non-consanguineous marriage, with a normal birth and developmental history presented at the outpatient clinic of the Quirino Memorial Medical Center (QMMC) due to one week history of recurrent episodes of seizures described as the sudden loss of muscle tone followed by stiffening of extremities and upward rolling of eyeballs. These events occurred more than 10 times per day lasting for approximately 10 to 15 seconds. The events were usually followed by post-ictal confusion and occasional urinary incontinence. Past medical history revealed that the patient received primary measles vaccination at nine months of age but still developed active measles infection at the age of two years.

**Figure 1:**
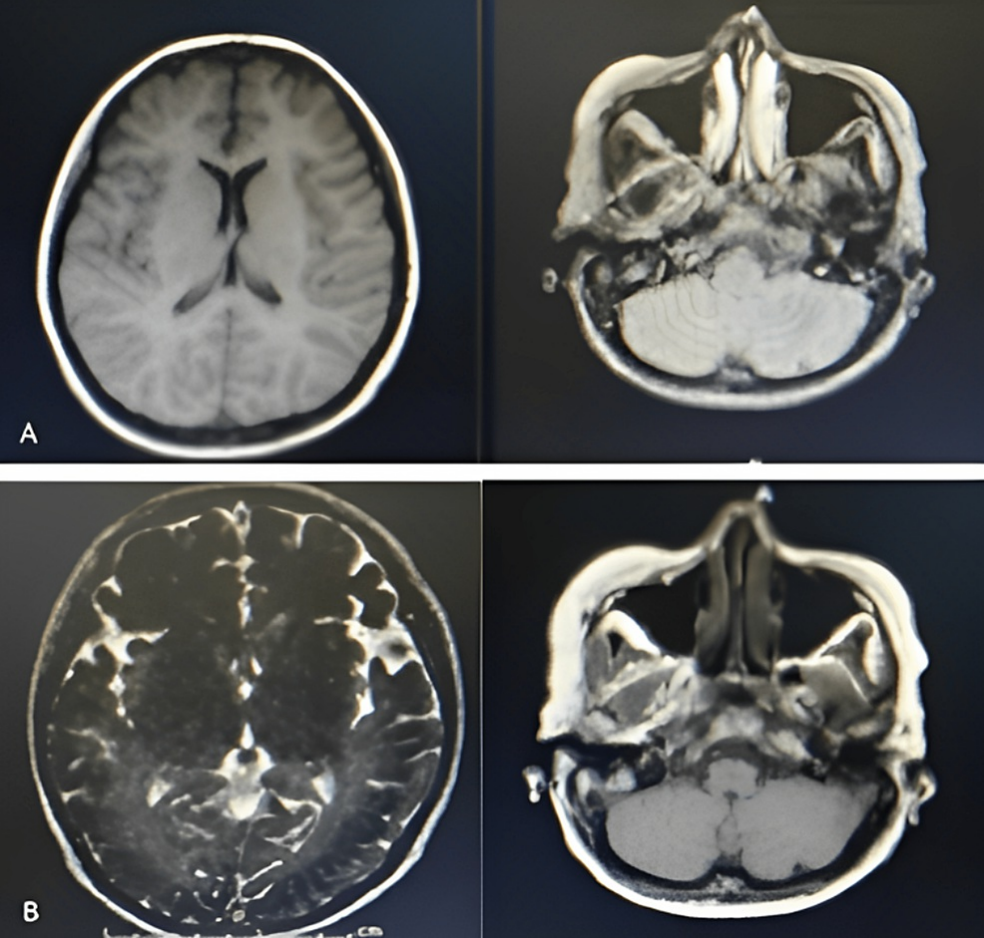
T1 (A) and T2 weighted (B) cranial MRI on the axial view taken during the patient’s first hospital admission showing chronic lacunar infarcts on the right frontal and left cerebellar lobes. MRI: magnetic resonance imaging

Physical and neurologic examinations on admission were unremarkable. A routine electroencephalography (EEG) performed showed normal findings with no electroclinical events noted. A cranial magnetic resonance imaging (MRI) with contrast revealed chronic small infarcts in the left inferior cerebellum and right anterior frontal lobes (Figure 1).

The patient was started on topiramate 450 mg/day and clonazepam 1 mg/day, however, the seizures persisted at 12 to 19 episodes each day lasting 12 to 20 seconds. Levetiracetam 2000 mg/day and lacosamide 100 mg/day were added which only decreased the seizure frequency but did not afford complete seizure cessation.

One month after, the patient developed oral ulcers associated with the malar rash formation and erythematous, papulomacular eruptions in the skin. An autoimmune workup was done showing an elevated ANA ratio of 1:2.7 and an anti-dsDNA level of 16.1 IU/mL (positive >15 IU/mL). The HIV serology, anticardiolipin immunoglobulin G (IgG), and immunoglobulin M (IgM) were negative. SLE with neuropsychiatric features was entertained. Prednisone 60 mg/day was given for one month and shifted to methylprednisolone 8 mg/day. There was an eventual resolution of the rash and a reduction of seizures to three to four episodes per day.

Nine months into the course of illness, the patient developed behavioral changes described as escalating irritability, incessant crying, and the presence of visual and auditory hallucinations. A lumbar puncture was performed to rule out central nervous system (CNS) infection showing normal cerebrospinal fluid (CSF) glucose, protein, and cell counts. Quetiapine 25 mg/day was added to the treatment regimen, however, it did not offer a resolution of symptoms.

During the next four months, there was a decline in the patient’s speech and motor faculties. The verbal output drastically decreased to one to three words and necessitated assistance with ambulation and other activities of daily living. In addition to the generalized tonic-clonic seizures, the patient also developed generalized myoclonic jerks involving the bilateral upper and lower extremities occurring in clusters several times per day. Antiseizure medications were revised to clonazepam 0.5 mg/day, levetiracetam 1500 mg/day, and lamotrigine 100 mg/day. Methylprednisolone was discontinued to rule out steroid-induced psychosis.

**Figure 2:**
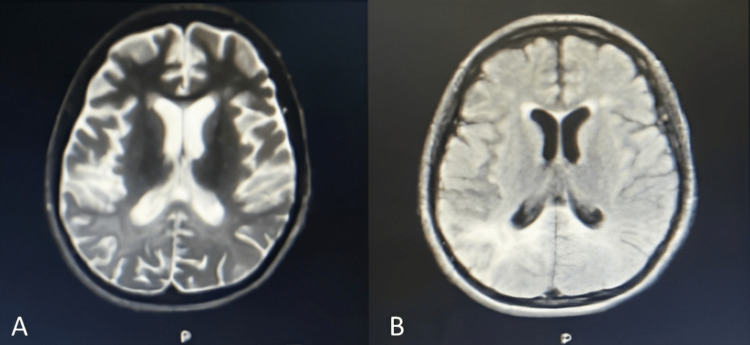
Cranial MRI as seen on axial view showing hyperintensities in the cerebral cortical, subcortical, and periventricular white matter changes on the bilateral parietooccipital and temporal areas on T2 weighted (A) and FLAIR (B) sequences. MRI: magnetic resonance imaging; FLAIR: fluid-attenuated inversion recovery

**Figure 3:**
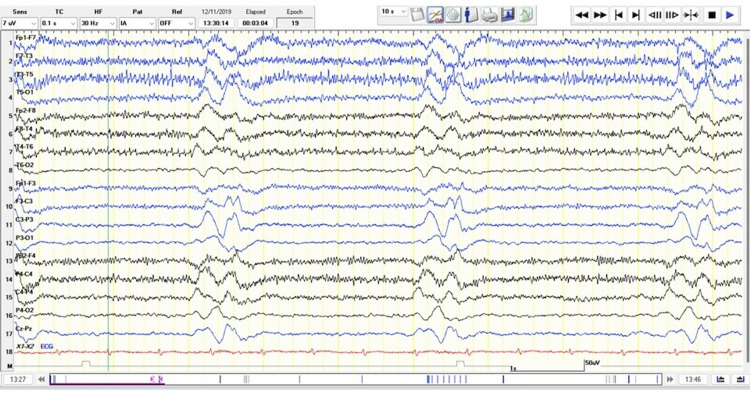
Follow-up EEG displaying frequent periodic bursts of generalized periodic slow wave activity (Radermecker complexes) correlated with generalized myoclonic jerks. There is diffuse slowing of the background activity in the alpha-theta range. EEG: electroencephalography

Despite the adjustments made, there was further deterioration in language, cognitive, and motor skills with loss of verbal output, expression limited to mostly crying at night, and occasional smiling. The patient became bedridden with episodic flexion of the extremities and head turning to the left with neck rigidity. There was also an increased frequency of generalized myoclonic jerks occurring repetitively throughout the day. The clinical progress prompted further investigation. During the latest admission, a follow-up cranial MRI with contrast taken revealed postictal changes involving the cortical, subcortical regions and periventricular white matter changes with a dominant presentation in the parietooccipital and temporal areas (Figure 2). A repeat EEG showed mixed frequency slowing of the background in the alpha-theta range with frequent bursts of periodic generalized slow wave complexes coinciding with generalized myoclonic jerks (Figure 3). An elevated Rubeola virus IgG was found on the patient’s cerebrospinal fluid at 5000.00 mIU/mL. The latest findings led to a consideration of SSPE on top of concurrent SLE. Antiseizure medications were adjusted by increasing levetiracetam to 2500 mg/day and clonazepam to 1 mg/day. Valproic acid at 1000 mg/day was also added to which the myoclonic jerks lessened from 17 to 20 episodes per day to four to seven per day. Methylprednisone was resumed at 16 mg/day and hydroxychloroquine at 200 mg/day was started for the SLE. The modifications controlled the SLE disease activity and the generalized tonic-clonic seizures; however, episodic generalized myoclonic jerks persisted. To date, the patient remained dependent on all activities of daily living and self-care needs. Referral to rehabilitation medicine was done and a percutaneous endoscopic gastrostomy tube was inserted for feeding for supportive care.

## Discussion

SSPE is a rare progressive, fatal neurological disorder of children and young adults that affects the CNS caused by a persistent existence of a defective measles virus seen in immunocompetent persons [6,7]. Patients with a history of measles infections usually at the age of less than two years are at great risk to develop SSPE. The manifestations of this condition usually appear seven to 10 years after the active infection [1]. The clinical manifestation of SSPE usually begins with personality and behavioral changes, followed by a progressive decline in motor function including myoclonic jerks, involuntary movements, and focal and/or generalized seizures. Eventually, rigidity, extrapyramidal symptoms, and unresponsiveness that lead to a vegetative or comatose state ensue [1,5,8]. Dyken's modified criteria is a tool to aid in diagnosing SSPE and includes clinical history, elevated CSF measles antibody titers as major markers, typical EEG findings, increased CSF IgG, brain biopsy, and special molecular diagnostic tests to identify measles virus mutated genome as minor markers [5]. A minimum of two major and one minor criterion were typically required for diagnosis, of which the patient fulfilled the following: an elevated cerebrospinal fluid measles antibody titer, the temporal evolution of the clinical manifestations, and generalized periodic complexes on EEG.

SLE is a chronic multisystemic autoimmune disease presenting a diverse range of clinical features of variable prognosis depending on organ involvement and disease activity [9]. Genetic, environmental, and hormonal factors play a role in its pathogenesis. The incidence and prevalence vary worldwide. In the Asia-Pacific regions, the numbers ranged from 0.9 to 3.1 and 4.3 to 45.3 per 100,000 respectively [10]. The age of onset is usually between 16 and 55 years with a female preponderance. Diagnosis is clinical, supported by laboratory findings including serologic assays [11]. The interplay of cognitive dysfunction, psychosis, and multiple seizure episodes in association with positive ANA and anti-dsDNA supported the diagnosis of SLE with neuropsychiatric features. The patient’s progressive neurologic decline while on immunosuppressive therapy and persistent episodes of auditory hallucinations impelled the clinician to delve into evaluating other possible conditions, which include corticosteroid-induced psychosis, CNS infection, and SSPE. The absence of microorganisms and polymorphonuclear cells in the cerebrospinal fluid studies ruled out an active CNS infection. The patient also showed no improvement in cognition even with the incorporation of antipsychotics and discontinuation of steroid therapy hence, glucocorticoid-induced psychosis was less likely.

A history of measles infection triggers autoreactive T and B cells through molecular mimicry resulting in a structural similarity between microbial peptides and self-antigens. Autoimmune reactions may result from the activation of several T-cells with different antigenic specialties may initiate increased cytokine production. This mechanism may lead to an autoreactive or memory T-cell expansion, that may facilitate the development of autoimmune diseases such as SLE [12].

Normally, a patient’s immune responses are stimulated during measles infection to enhance viral load clearance, clinical improvement, and establishment of long-term immunity [13]. Inoue et al. investigated the correlation of interleukin 4 (IL-4) gene susceptibility to SSPE since IL-4 promoter 589 C/T, IL-4 receptor alpha-chain polymorphism has been associated with SLE, which is a disorder of the Th1/Th2 balance [14]. Measles is associated with Th1 activation (increased IFN-y and IL-2 levels) before and during the rash phase, followed by preferential Th2 activation (decreases IFN-y and IL-2 levels and increases IL-4 levels) during the convalescent phase. This Th2 response leads to excessive anti-measles antibody production [2,14]. In some individuals however, the measles virus is incompletely eradicated possibility as a consequence of a reduced cellular immune response and an elevated humoral immune response, leading to the persistence of the virus in the host which leads to the subsequent risk of developing SSPE. The immaturity of the immune system of younger individuals exposed to the virus may explain the propensity of SSPE development in this subset of patients [15]. Coinfection with HIV has been reported in observational case series which may also increase the risk for its development and influence its clinical presentation [5].

There is limited data regarding the possible influence of a coexisting autoimmune disease such as SLE in the development and clinical course of SSPE. Utermohlen et al. emphasize that patients known to have SLE showed specific depression of direct leukocyte migration inhibition of measles antigen as compared with controls, while normal inhibition to migration occurred with rubella and parainfluenza type 1 [16]. The experiment suggests that the presence of SLE can lower the body’s host immune responses and may contribute to the incomplete elimination of the measles virus in the body or the possible reactivation of a latent measles virus infection. Moreover, a case-control study done by Maritsi et al. showed that there is an accelerated antibody loss against measles in children with childhood SLE during the disease course [17]. These processes also explain the contribution of SLE in the development reactivation of latent measles virus leading to SSPE such as in the case of the patient.

There is no effective treatment available as of writing [1,6,7,18,19]. The administration of amantadine and inosine pranobex was found by some investigators to lead to improvement and prolonged survival, but these effects have not been corroborated [6,7,18].

## Conclusions

The key mechanisms highlighted in this case report explain the theoretical association between the coexistence of SSPE and SLE. The development of SSPE may be due to the downregulation of host-immune responses that resulted in incomplete measles viral clearance. This gave way to the persistence of immune complexes, thereby facilitating the development of autoimmunity and the potential development of SLE. The presence of SLE eventually leads to the downregulation of T-cell responses and accelerated loss of antibodies previously formed against the measles virus, thereby providing an avenue for the development of a latent measles infection and evolution to SSPE. This case report emphasizes that it is vital to search for a history of measles infection in patients diagnosed with SLE manifesting with neuropsychiatric manifestations and to include SSPE in the workup once these symptoms are manifested. Treatments are not curative and the families of patients with SSPE have a lot of physical, psychological, and economical stresses to endure requiring a great deal of external support.

To date, from a thorough review of the literature, this case belongs to a list of a few well-documented SSPE in the Philippines and perhaps the first published case with a concurrent diagnosis of SLE. We recommend further studies and documentation of similar cases to widen our understanding of the relationship between SSPE and SLE.
